# Temporal change in multimorbidity prevalence, clustering patterns, and the association with mortality: findings from the China Kadoorie Biobank study in Jiangsu Province

**DOI:** 10.3389/fpubh.2024.1389635

**Published:** 2024-04-18

**Authors:** Hao Yu, Ran Tao, Jinyi Zhou, Jian Su, Yan Lu, Yujie Hua, Jianrong Jin, Pei Pei, Canqing Yu, Dianjianyi Sun, Zhengming Chen, Liming Li, Jun Lv

**Affiliations:** ^1^Department of Epidemiology and Biostatistics, School of Public Health, Peking University, Beijing, China; ^2^Department of Noncommunicable Chronic Disease and Prevention, Jiangsu Provincial Center for Disease Control and Prevention, Nanjing, China; ^3^Department of Noncommunicable Chronic Disease Control and Prevention, Suzhou City Center for Disease Control and Prevention, Suzhou, China; ^4^Department of Noncommunicable Chronic Disease Control and Prevention, Wuzhong District Center for Disease Control and Prevention, Suzhou, China; ^5^Peking University Center for Public Health, Epidemic Preparedness and Response, Beijing, China; ^6^Key Laboratory of Epidemiology of Major Diseases, Peking University, Ministry of Education, Beijing, China; ^7^Clinical Trial Service Unit and Epidemiological Studies Unit, Nuffield Department of Population Health, University of Oxford, Oxford, United Kingdom

**Keywords:** multimorbidity, cluster analysis, cohort study, prevalence, mortality

## Abstract

**Objectives:**

The characteristics of multimorbidity in the Chinese population are currently unclear. We aimed to determine the temporal change in multimorbidity prevalence, clustering patterns, and the association of multimorbidity with mortality from all causes and four major chronic diseases.

**Methods:**

This study analyzed data from the China Kadoorie Biobank study performed in Wuzhong District, Jiangsu Province. A total of 53,269 participants aged 30–79 years were recruited between 2004 and 2008. New diagnoses of 15 chronic diseases and death events were collected during the mean follow-up of 10.9 years. Yule's Q cluster analysis method was used to determine the clustering patterns of multimorbidity. A Cox proportional hazards model was used to estimate the associations of multimorbidity with mortalities.

**Results:**

The overall multimorbidity prevalence rate was 21.1% at baseline and 27.7% at the end of follow-up. Multimorbidity increased more rapidly during the follow-up in individuals who had a higher risk at baseline. Three main multimorbidity patterns were identified: (i) cardiometabolic multimorbidity (diabetes, coronary heart disease, stroke, and hypertension), (ii) respiratory multimorbidity (tuberculosis, asthma, and chronic obstructive pulmonary disease), and (iii) mental, kidney and arthritis multimorbidity (neurasthenia, psychiatric disorders, chronic kidney disease, and rheumatoid arthritis). There were 3,433 deaths during the follow-up. The mortality risk increased by 24% with each additional disease [hazard ratio (HR) = 1.24, 95% confidence interval (CI) = 1.20–1.29]. Compared with those without multimorbidity at baseline, both cardiometabolic multimorbidity and respiratory multimorbidity were associated with increased mortality from all causes and four major chronic diseases. Cardiometabolic multimorbidity was additionally associated with mortality from cardiovascular diseases and diabetes, with HRs of 2.64 (95% CI = 2.19–3.19) and 28.19 (95% CI = 14.85–53.51), respectively. Respiratory multimorbidity was associated with respiratory disease mortality, with an HR of 9.76 (95% CI = 6.22–15.31).

**Conclusion:**

The prevalence of multimorbidity has increased substantially over the past decade. This study has revealed that cardiometabolic multimorbidity and respiratory multimorbidity have significantly increased mortality rates. These findings indicate the need to consider high-risk populations and to provide local evidence for intervention strategies and health management in economically developed regions.

## 1 Introduction

The rapid aging of populations worldwide is increasing the prevalence of the coexistence of two or more chronic diseases simultaneously. Such multimorbidity brings heavy burdens and significant challenges to patients, caregivers, medical professionals, and healthcare systems (1–3). However, evidence-based decision-making regarding multimorbidity conditions remains problematic.

Epidemiological studies have demonstrated variations in the prevalence of multimorbidity between populations exhibiting distinct characteristics, such as its prevalence being higher among older adults and females (4). There are also regional variations in the clustering patterns of multimorbidity. Results from the COURAGE and SAGE programs have identified common clustering patterns in multiple countries, including cardio-respiratory, metabolic, and mental-articular (5). A review involving over 70 million patients from 12 countries indicated that the most frequent combination of multimorbidity was hypertension and osteoarthritis, followed by various combinations of cardiovascular diseases (6). A cross-sectional finding from 10 regions in China revealed that the most common patterns of multimorbidity aggregation nationwide involved cardiometabolic, respiratory, gastrointestinal, hepatorenal, mental, and arthritis conditions (7). These regional disparities not only reflect the impacts of lifestyle and environment on health but also highlight the importance of formulating health policies and disease prevention strategies tailored to specific contexts.

There is evidence that multimorbidity is influenced by both the number of diseases present and the specific multimorbidity combinations (8, 9). The Healthy China 2030 plan aims to increase life expectancy and reduce premature mortality from four major chronic diseases: cardiovascular diseases, cancer, respiratory diseases, and diabetes. The current understanding of the association between multimorbidity and the causes of death from four major chronic diseases is inadequate. Most previous multimorbidity studies have been cross-sectional, with insufficient data available from cohort studies. Moreover, the previous evidence primarily comes from Western countries, so high-quality, region-specific evidence still needs to be obtained for the Chinese population.

This study investigated a natural population cohort of 53,269 individuals from the China Kadoorie Biobank (CKB) study performed in Wuzhong District, Suzhou City, Jiangsu Province, in eastern China. The aim was to compare the population distribution of multimorbidity prevalence in the cohort population between baseline and the end of follow-up, identify frequent cluster patterns of multimorbidity in the cohort population, and determine the prospective association of multimorbidity with mortality from all causes and four major chronic diseases.

## 2 Materials and methods

### 2.1 Study population

The prospective cohort study was conducted in Wuzhong District, an economically developed region in southern Jiangsu Province, between 2004 and 2008. As part of the CKB study, further details have been described elsewhere (10). Briefly, 53,269 participants aged 30–79 years with valid baseline information were recruited and followed up for a mean of 10.9 years. The general demographic information, lifestyle behavior, and self-reported history of chronic diseases were collected through face-to-face interviews by unified trained staff. Physical measurements and biochemical tests were applied using calibrated instruments. All participants were followed up through the linkage to the death and disease surveillance systems, national health insurance claim database, and separate active confirmations to obtain hospital admission and death information. Participants who were unable to obtain information were recorded as having a loss of follow-up. By the end of 2017, 49,391 participants were remaining due to 3,433 deaths and 445 being lost to follow-up (0.84%). The study was approved by the ethics committees of both Chinese and British research institutions. All participants provided written informed consent.

### 2.2 Definitions of the chronic disease status

The baseline chronic disease was determined by a comprehensive assessment performed using questionnaire surveys, physical examinations, and biochemical tests. A total of 15 chronic diseases were included in the baseline multimorbidity analyses, which were hypertension, diabetes, chronic obstructive pulmonary disease (COPD), chronic liver disease (CLD), gallbladder disease, psychiatric disorders, coronary heart disease (CHD), stroke, cancer, tuberculosis, rheumatoid arthritis (RA), asthma, peptic ulcer, chronic kidney disease (CKD), and neurasthenia.

Hypertension was defined as a self-reported clinical diagnosis of hypertension, recent use of antihypertensive drugs, or two blood pressure measurements with mean systolic blood pressure ≥ 140 mmHg and/or mean diastolic blood pressure ≥ 90 mmHg. Diabetes was also self-reported based on recent taking of hypoglycemic agents, or tests with random plasma glucose ≥ 11.1 mmol/L or fasting plasma glucose ≥ 7.0 mmol/L. COPD was defined as self-reported chronic bronchitis, emphysema, or a lung function test ratio of forced expiratory volume in the first second to forced vital capacity with a value <0.7. CLD was defined as self-reported chronic hepatitis, cirrhosis, or positivity for the hepatitis B surface antigen. Gallbladder disease was defined as self-reported gallstones or cholecystitis. A psychiatric disorder was defined as a self-reported diagnosis of a psychological disorder by a medical specialist. In addition, if the subject had experienced emotional depression and anxiety for more than 2 weeks during the past year, they completed a WHO short version of the Composite International Diagnostic Interview Form and were evaluated according to the Diagnostic and Statistical Manual of Mental Disorders.

The chronic diseases present at the end of the follow-up were determined based on the chronic disease history collected at baseline, supplemented by new diagnoses during the follow-up period. Diagnostic information was obtained in multiple ways, including local passive disease surveillance systems, medical insurance records, and active monitoring. Chronic diseases were identified using the following codes in the tenth revision of the International Classification of Disease (ICD-10): hypertension (H35.0, I10–I12, I15.0, I15.1, I15.9, I67.4, O13), diabetes (E10–E14), stroke (I60, I61, I63, I64), cancer (C00–C97), COPD (J41–J44), CHD (I20–I25), tuberculosis (A15, A16), RA (M05, M06, M08.0, M45), asthma (J45, J46), CLD (B18, B19, K72.1, K72.9, K73, K74, K75.2–K75.4), gallbladder disease (K80, K81), peptic ulcer (K25–K28), CKD (N02, N03, N07, N11, N18), psychiatric disorders (F03, F20.4, F06.4, F25.1, F25.2, F31–F33, F34.1, F40.8, F40.9, F41, F60.6, F92.0, F92.8, F93.0–F93.2, F93.8,), and neurasthenia (F48.0).

### 2.3 Assessment of covariates

The relevant covariates included sociodemographic characteristics (age, sex, education level, marital status, occupational status, and household income), lifestyle behaviors (smoking status, alcohol consumption, physical activity, and dietary intake frequency of fresh vegetables, fruits, and meat), medical history (whether first-degree relatives had experienced a stroke, myocardial infarction, diabetes, or cancer, as well as the menopausal status for females only). Body mass index (BMI) was calculated as the weight in kilograms divided by the square of the height in meters. Metabolic equivalent task (MET) values were applied to quantify the daily level of physical activities.

### 2.4 Ascertainment of death outcomes

Information about deaths was obtained from the Jiangsu Province Population Death Registration System, ensuring high-quality data that was complete and accurate. Causes of death indicated in official death certificates were augmented by reviewing medical records if needed. Regular investigations into death underreporting were conducted as a supplement to routine reports. Trained professionals identified the underlying causes of death and classified them according to ICD-10 into the following categories: all causes (A00–Y89), cardiovascular diseases (I00–I99), cancer (C00–C97), respiratory diseases (J00–J98), diabetes (E10-E14), and four major chronic diseases (C00–C97, I00–I99, E10–E14, J30–J98).

### 2.5 Statistical analyses

The numbers of diseases at baseline and the end of follow-up were quantified according to the baseline characteristics. An unconditional logistic regression model was used to compare the differences in multimorbidity prevalence among different sexes and age groups.

Multimorbidity patterns were extracted based on the principle of hierarchical cluster analysis. Yule's Q cluster analysis method was used to detect the distances between different diseases, with a smaller distance coefficient indicating a more significant correlation between the two diseases. Dendrograms from cluster analysis were used to display the classification results. The observed prevalence, expected prevalence, and their ratios were calculated for each multimorbidity pattern. A ratio higher than 1 indicates an association between diseases, with a larger ratio indicating a stronger association. The expected prevalence rate was obtained by multiplying the prevalence of each disease within each combination of multimorbidity.

A Cox proportional hazards regression model was used to estimate the association of multimorbidity at baseline with the risks of mortality from all causes and four major chronic diseases, yielding hazard ratios (HRs) and 95% confidence intervals (CIs). The proportional hazards assumption was confirmed using the Schoenfeld residual method and log-log plot. Multivariable models were adjusted for the baseline age, sex, education level, marital status, occupational status, household income, smoking status, alcohol consumption, physical activity, intake frequency of fresh vegetables, fruits, and meat, menopausal status (only in females), and family history of chronic diseases.

A linear trend test was used to explore the prospective association between the number of multimorbidities and the risk of death. The number of diseases was included in the Cox regression model as a continuous variable, and a linear trend was considered present in the correlation when *P* < 0.05.

Sensitivity analysis was used to examine the stability of the results. Continuous BMI and waist circumference (WC) were added to the model to reexamine the relationships between the numbers of chronic diseases, multimorbidity clustering patterns, combinations, and mortality from all causes and four major chronic diseases.

Subgroup analyses used a likelihood-ratio test to explore whether the following dichotomized potential risk factors modified the association between multimorbidity and mortality: sex (male, female), age (<60, ≥60, years), education level (primary school and below, middle school and above), marital status (married, unmarried), smoking status (not smokers included non-smokers and seldom smokers, daily smokers included current smokers and former smokers), alcohol consumption (non-excessive drinkers included non-drinkers, seldom drinkers, and daily consumption <30 grams, excessive drinkers included people who quit drinking and daily consumption ≥30 grams), physical activity (MET divided into low, middle, and high groups), menopausal status (premenopause, postmenopause), overweight or obesity (BMI ≥24 kg/m^2^, WC ≥85 cm for males, or ≥80 cm for females), and family history of chronic diseases (yes, no).

The statistical analyses were performed with Stata software (version 15.0). All *P-*values were two-sided, and *P* < 0.05 was considered to indicate statistical significance.

## 3 Results

### 3.1 Prevalence rate of chronic diseases at baseline and the follow-up

The prevalence of multimorbidity at the end of follow-up was 27.7%, which was 6.6% higher than the baseline rate of 21.1%. Participants with the specific characteristics showed a higher prevalence of multimorbidity and faster growth during the follow-up, such as adults aged ≥60 years; having a low education level or low household income; being unmarried or a non-worker, non-farmer, or excessive drinkers; having a low physical activity or not frequently consuming vegetables, fruits, or meat; being overweight or obese; or having a family history of chronic diseases. There was no statistically significant difference between males and females or between non-smokers and daily smokers (Supplementary Table S1).

### 3.2 Cluster patterns at baseline and the end of follow-up

**Figure 1:**
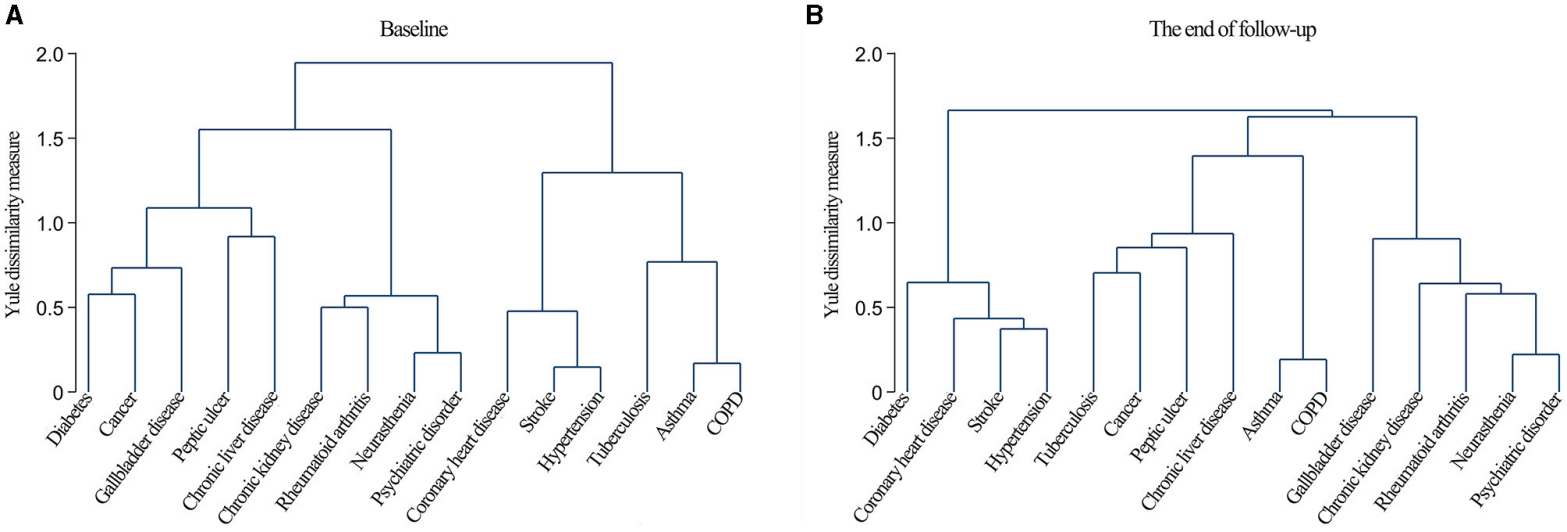
Dendrograms of cluster analysis showing the aggregation of chronic diseases at baseline **(A)** and the end of follow-up **(B)**.

The dendrograms obtained from the cluster analysis (Figure 1) indicated that the chronic diseases at baseline mainly clustered into four patterns: cardiometabolic multimorbidity (CHD, stroke, and hypertension), respiratory multimorbidity (tuberculosis, asthma, and COPD), mental, kidney and arthritis multimorbidity (neurasthenia, psychiatric disorders, CKD, and RA), and diabetes, cancer, and gallbladder multimorbidity (diabetes, cancer, and gallbladder disease).

The clustering patterns at the end of the follow-up could also be categorized into four types: cardiometabolic multimorbidity (diabetes, CHD, stroke, and hypertension), respiratory multimorbidity (asthma and COPD), mental, kidney, and arthritis multimorbidity (neurasthenia, psychiatric disorders, CKD, RA, and gallbladder disease), and tuberculosis and cancer multimorbidity (tuberculosis, cancer, peptic ulcers, and CLD).

Finally, based on the similar clustering patterns exhibited at baseline and the end of follow-up, as well as their potential pathophysiological mechanisms, we extracted three clustering patterns: cardiometabolic multimorbidity (CHD, stroke, hypertension, and diabetes), respiratory multimorbidity (tuberculosis, asthma, and COPD), and mental, kidney, and arthritis multimorbidity (neurasthenia, psychiatric disorders, CKD, and RA).

Combinations of these three clustering patterns were compared between the baseline and the end of the follow-up. The combination of hypertension + diabetes was the most commonly observed both at baseline and the end of follow-up, followed by the combinations of hypertension + stroke and hypertension + CHD. Within the clustering pattern of comorbidities of respiratory multimorbidity, the prevalence rates at baseline and the end of follow-up were higher for COPD + asthma, followed by COPD + tuberculosis. Within the clustering pattern of mental, kidney, and arthritis multimorbidity, the most frequent combination was neurasthenia + psychiatric disorder, followed by neurasthenia + RA (Table 1).

**Table 1:** Combinations of multimorbidity at baseline and the end of follow-up.

**Multimorbidity**	**Baseline**				**The end of follow-up**			
	**No. of pairs**	**Observed prevalence (%)** ^a^	**Expected prevalence (%)** ^b^	**Ratio O/E**	**No. of pairs**	**Observed prevalence (%)** ^a^	**Expected prevalence (%)** ^b^	**Ratio O/E**
**Cardiometabolic multimorbidity**								
Hypertension + diabetes	1,719	3.23	2.10	1.54	2,329	4.72	3.76	1.26
Hypertension + stroke	331	0.62	0.36	1.72	1,242	2.51	1.98	1.27
Hypertension + CHD	313	0.59	0.44	1.34	678	1.37	1.20	1.14
Hypertension + diabetes + stroke	62	0.12	0.02	6.00	356	0.72	0.19	3.79
Hypertension + diabetes + CHD	61	0.11	0.02	5.50	180	0.36	0.12	3.00
Diabetes + CHD	10	0.02	0.06	0.33	48	0.10	0.30	0.33
Diabetes + stroke	5	0.01	0.05	0.20	99	0.20	0.49	0.41
**Respiratory multimorbidity**								
COPD + asthma	231	0.43	0.09	4.78	230	0.46	0.10	4.60
COPD + tuberculosis	134	0.25	0.13	1.92	123	0.25	0.14	1.79
COPD + tuberculosis + asthma	9	0.02	0.001	20.00	7	0.01	0.001	10.00
**Mental, kidney and arthritis multimorbidity**								
Neurasthenia + psychiatric disorders	107	0.20	0.04	5.00	116	0.23	0.04	5.75
Neurasthenia + RA	71	0.13	0.04	3.25	64	0.13	0.04	3.25
Psychiatric disorders + RA	36	0.07	0.04	1.75	43	0.09	0.05	1.80
Psychiatric disorders + CKD	19	0.04	0.01	4.00	22	0.04	0.02	2.00
Neurasthenia + CKD	14	0.03	0.01	3.00	14	0.03	0.02	1.50
RA + CKD	14	0.03	0.01	3.00	15	0.03	0.02	1.50
Neurasthenia + psychiatric disorders + RA	7	0.01	0.001	10.00	7	0.01	0.001	10.00

CHD, Coronary heart disease; COPD, Chronic obstructive pulmonary disease; RA, Rheumatoid arthritis; CKD, Peptic ulcer, chronic kidney disease. ^a^Observed prevalence was accurate to 0.01%, results < 0.01 were not displayed. ^b^Expected prevalence was obtained by multiplying the independent prevalence of each disease within the combination. Expected prevalence was accurate to 0.01%, results < 0.01 were not displayed.

### 3.3 Association between the number of chronic diseases and mortality

The 3,433 deaths that occurred during the follow-up comprised 1,687 cases of cancer, 910 cases of cardiovascular diseases, 219 cases of respiratory diseases, and 65 cases of diabetes. The mortality risk increased by 24% with each additional disease (HR = 1.24, 95% CI = 1.20–1.29). There was a significant dose-response relationship for mortality from all causes and independently for cardiovascular diseases, cancer, respiratory diseases, and diabetes (*P* < 0.05). Compared with the baseline status of without multimorbidity, multimorbidity significantly increased the risks of mortality from all causes, four major chronic diseases, and each chronic disease (*P* < 0.05). Diabetes mortality showed the greatest increase, with an HR of 6.64 (95% CI = 3.64–12.11) (Table 2).

**Table 2:** Association between the number of chronic diseases and mortality.

**Causes**	**No. of diseases**	**No. of deaths**	**Mortality^a^**	**HR (95%CI)** ^**b**^		
				**Model 1**	**Model 2**	**Model 3**
**All causes**						
	0–1	2,020	4.37	1.00	1.00	1.00
	≥2	1,413	11.84	1.43 (1.33–1.53)	1.44 (1.34–1.54)	1.41 (1.31–1.51)
	Trend for numbers^†^			1.24 (1.20–1.29)	1.26 (1.21–1.30)	1.24 (1.20–1.29)
**Four major chronic Diseases**						
	0–1	1,667	3.61	1.00	1.00	1.00
	≥2	1,188	9.95	1.42 (1.31–1.53)	1.43 (1.32–1.55)	1.40 (1.29–1.51)
	Trend for numbers			1.25 (1.20–1.30)	1.27 (1.22–1.32)	1.25 (1.20–1.31)
**Cardiovascular Disease**						
	0–1	480	1.04	1.00	1.00	1.00
	≥2	430	3.60	1.59 (1.39–1.81)	1.57 (1.37–1.80)	1.53 (1.33–1.75)
	Trend for numbers			1.39 (1.29–1.49)	1.39 (1.29–1.50)	1.37 (1.27–1.47)
**Cancer**						
	0–1	1,084	2.35	1.00	1.00	1.00
	≥2	603	5.05	1.20 (1.08–1.33)	1.22 (1.10–1.35)	1.20 (1.08–1.33)
	Trend for numbers			1.12 (1.06–1.18)	1.14 (1.07–1.20)	1.13 (1.07–1.19)
**Respiratory Disease**						
	0–1	100	0.22	1.00	1.00	1.00
	≥2	119	1.00	1.98 (1.51–2.60)	2.06 (1.56–2.70)	1.96 (1.49–2.59)
	Trend for numbers			1.47 (1.27–1.70)	1.53 (1.32–1.77)	1.49 (1.28–1.73)
**Diabetes**						
	0–1	15	0.03	1.00	1.00	1.00
	≥2	50	0.42	7.04 (3.87–12.80)	7.06 (3.88–12.83 )	6.64 (3.64–12.11)
	Trend for numbers			2.91 (2.19–3.86)	3.01 (2.26–4.02)	2.93 (2.19–3.92)

HR, Hazard ratios; CI, Confidence intervals. ^†^The HR of mortality increased with every additional disease. ^a^The mortality was calculated by dividing the number of deaths by the number of follow-up years and multiplying by 1000. ^b^Model 1: Adjusted for age (years); Model 2: Adjusted for general demographic characteristics at baseline, including age (years), sex (male, female), education level (no formal school, primary school, middle school, high school, junior college, university and above), marital status (married, widowed, separated/divorced, unmarried), household income (< 20000 yuan, ≥20000 yuan), occupation (agricultural labors, workers, administrative personnel, technician, sales and service staff, retirees, household workers, private owners, unemployed and other individuals); Model 3: Based on model 2, an additional of smoking status (non-smoker, former smoker who had stopped due to illness, former smoker who had stopped for other reasons, current smoker with 1–19, or ≥ 20 cigarettes/day), alcohol consumption (non-drinker, ex-regular drinker, weekly but not daily drinker, daily drinker with 1–14 g, 15–29 g, 30–59 g, ≥60 g pure alcohol/day), physical activity (MET h/d), intake frequency of meat, fresh vegetables and fruits (daily, 4–6 days, 1–3 days/week, several times per month, never or seldom), family history of stroke, myocardial infarction, diabetes, and cancer (yes or no, and adjusted for above family histories when analyzing association with mortality from all causes and four major chronic diseases, adjusted for family history of stroke and myocardial infarction when cardiovascular disease was related, adjusted for the family history of cancer when cancer was related; and adjusted for the family history of diabetes when diabetes was related, unadjusted for family history when analyzing respiratory system diseases.

### 3.4 Association between multimorbidity patterns and mortality

We analyzed the association between baseline multimorbidity patterns and mortality, excluding patterns corresponding to deaths below 5 cases. After adjustment, compared with the baseline status of non-multimorbidity, the presence of cardiometabolic multimorbidity increased the risks of mortality from all causes, four major chronic diseases, cardiovascular disease, and diabetes. Respiratory multimorbidity increased the risks of mortality from all causes, four major chronic diseases, and respiratory diseases. The mental, kidney, and arthritis multimorbidity did not significantly impact mortality (*P* > 0.05) (Table 3).

**Table 3:** Association between multimorbidity patterns and mortality.

**Causes**	**Patterns**	**No. of multimorbidity**	**No. of deaths**	**Mortality^a^**	**Model 1 HR (95%CI)**	**Model 2 HR (95%CI)**	**Model 3 HR (95%CI)^b^**
**All causes**							
	Non- multimorbidity	42,047	2,020	4.37	1.00	1.00	1.00
	Cardiometabolic multimorbidity	2,524	425	16.34	1.76 (1.59–1.96)	1.81 (1.62–2.02)	1.78 (1.59–1.99)
	Respiratory multimorbidity	374	85	22.17	2.12 (1.71–2.64)	1.88 (1.51–2.34)	1.82 (1.46–2.27)
	Mental, kidney, and arthritis multimorbidity	279	20	6.50	0.96 (0.62–1.50)	1.21 (0.78–1.88)	1.22 (0.78–1.89)
**Four major chronic diseases**							
	Non- multimorbidity	42,047	1,667	3.61	1.00	1.00	1.00
	Cardiometabolic multimorbidity	2,524	366	14.07	1.80 (1.60–2.02)	1.87 (1.66–2.10)	1.83 (1.63–2.07)
	Respiratory multimorbidity	374	76	19.82	2.24 (1.77–2.82)	1.97 (1.56–2.49)	1.89 (1.50–2.40)
	Mental, kidney, and arthritis multimorbidity	279	14	4.55	0.80 (0.47–1.36)	1.03 (0.61–1.74)	1.02 (0.60–1.74)
**Cardiovascular disease**							
	Non-multimorbidity	42,047	480	1.04	1.00	1.00	1.00
	Cardiometabolic multimorbidity	2,524	174	6.69	2.64 (2.22–3.15)	2.77 (2.31–3.32)	2.64 (2.19–3.19)
	Respiratory multimorbidity	374	20	5.22	1.74 (1.11–2.73)	1.52 (0.96–2.38)	1.49 (0.94–2.35)
	Mental, kidney, and arthritis multimorbidity	279	6	1.95	1.16 (0.52–2.59)	1.33 (0.59–2.99)	1.30 (0.58–2.94)
**Cancer**							
	Non-multimorbidity	42,047	1,084	2.35	1.00	1.00	1.00
	Cardiometabolic multimorbidity	2,524	135	5.19	1.10 (0.91–1.31)	1.13 (0.94–1.35)	1.12 (0.93–1.35)
	Respiratory multimorbidity	374	27	7.04	1.35 (0.92–1.98)	1.21 (0.82–1.78)	1.16 (0.79–1.71)
	Mental, kidney and arthritis multimorbidity	279	7	2.28	0.63 (0.30–1.32)	0.82 (0.39–1.73)	0.80 (0.38–1.68)
**Respiratory disease** ^c^							
	Non-multimorbidity	42,047	100	0.22	1.00	1.00	1.00
	Cardiometabolic multimorbidity	2,524	16	0.61	1.11 (0.66–1.90)	1.17 (0.68–2.03)	1.18 (0.67–2.05)
	Respiratory multimorbidity	374	29	7.56	12.10 (7.94–18.44)	10.15 (6.56–15.70)	9.76 (6.22–15.31)
**Diabetes**							
	Non-multimorbidity	42,047	15	0.03	1.00	1.00	1.00
	Cardiometabolic multimorbidity	2,524	43	1.65	27.08 (14.49–50.59)	29.89 (16.00–55.82)	28.19 (14.85–53.51)

HR, Hazard ratios; CI, Confidence intervals. ^a^The mortality was calculated by dividing the number of deaths by the number of follow-up years and multiplying by 1000. ^b^Multivariable models were adjusted for the same covariates as those in Table 2. ^c^The number of deaths from respiratory diseases related to mental, kidney, and arthritis multimorbidity was below 5, and no relevant results were reported in the table. ^d^The number of deaths from diabetes related to respiratory multimorbidity or mental, kidney, and arthritis multimorbidity was below 5, and no relevant results were reported in the table.

Further analyses were performed to explore the associations between different combinations of multimorbidities and mortality. Compared with individuals without cardiometabolic multimorbidity at baseline, the combination of stroke + hypertension + diabetes experienced the greatest impact, with a 2.65-fold increase in all-cause mortality, a 3.13-fold increase in mortality from four major chronic diseases and a 6.47-fold increase in mortality from cardiovascular diseases. Only the combination of hypertension + diabetes was associated with diabetes mortality, with an HR of 43.78 (95% CI = 24.93–76.88) (Supplementary Table S2). Compared with the baseline status without respiratory multimorbidity, both combinations of COPD + asthma and COPD + tuberculosis resulted in increased risks of mortality from all causes, four major chronic diseases, and respiratory diseases. The combination of COPD + asthma showed relatively high risks of mortality from all causes, four major chronic diseases, and respiratory diseases, with HRs of 1.56 (95% CI = 1.17–2.09), 1.72 (95% CI = 1.27–2.33), and 9.23 (95% CI = 5.71–14.92), respectively (Supplementary Table S3).

### 3.5 Sensitivity analyses

After additional adjustments for BMI and WC, a dose-response relationship still existed between the number of chronic diseases and mortality (Supplementary Table S4). The patterns of cardiometabolic multimorbidity and respiratory multimorbidity were associated with increased risks of mortality from all causes and four major chronic diseases (*P* < 0.05) (Supplementary Table S5). In addition, the combination of stroke + hypertension + diabetes was consistently observed to have a substantial effect on increasing mortality (Supplementary Table S6).

### 3.6 Subgroup analyses

The subgroup analyses revealed that individuals < 60 years had a higher risk of mortality caused by multimorbidity. Married people were more likely to die from cardiovascular diseases due to multimorbidity. The association of multimorbidity with mortality from respiratory diseases and diabetes did not appear to be significantly influenced by demographic characteristics and lifestyles (*P* for interaction >0.05) (Supplementary Tables S7–S10). In addition, we further adjusted several common dietary factors, such as tea, spicy food, preserved vegetables, and eggs, with no significant changes in the results (not displayed).

## 4 Discussion

This prospective cohort study found that the multimorbidity prevalence rate increased from 21.1% at baseline to 27.7% at the end of follow-up. There were dose-response relationships between the number of chronic diseases with mortality from all causes and four major chronic diseases. The chronic diseases were mainly clustered into three patterns: cardiometabolic multimorbidity, respiratory multimorbidity, and mental, kidney, and arthritis multimorbidity. The occurrence of multimorbidity significantly increased the risks of mortality from all causes and four major chronic diseases.

Different studies have revealed marked variations in the prevalence of multimorbidity. A meta-analysis found a considerable fluctuation in the incidence of multimorbidity among the general population, ranging from 13.1% to 71.8% (11). The findings of a cross-sectional survey conducted in southern China involving 160,000 individuals indicated a prevalence rate of 11.1% (10.6% to 11.6%) (12). Differences in the definition of multimorbidity, measurement methods, the inclusion of various types of chronic diseases, and recruited populations with diverse characteristics may affect assessments of multimorbidity prevalence (13). It might therefore not be appropriate to compare the prevalence of multimorbidity in the present study with previous reports. In the current cohort, we observed that the prevalence of multimorbidity had increased by 6.6% over the past 10 years, representing an increase of nearly one-third.

Similar to most previous research findings, we observed that in addition to conventional factors such as age and sex, social and lifestyle factors also played essential roles in multimorbidity (14). Education level was considered as a surrogate of socioeconomic status. According to the National Health Interview Survey performed in the US, individuals with less than a high school education had an HR risk of 2.16 (95% CI = 2.07–2.26) relative to those with higher education levels (15). Thus, improving the education level of a population would potentially be effective at reducing or preventing multimorbidity. Unhealthy lifestyles are predisposing factors for various chronic diseases. The Jiangsu Nutrition Study discovered that great consumption of fruits, vegetables, and whole-grain products was associated with a reduced risk of multimorbidity (16). National Health and Nutrition Examination Surveys of US adults detected a negative correlation between the number of chronic conditions and daily physical activity (17). Furthermore, an increased number of unhealthy lifestyle factors was associated with a higher likelihood of multimorbidity (18). Therefore, it is necessary to implement comprehensive behavioral interventions to reduce the cumulative and excess detrimental effects of multiple unhealthy lifestyle factors on multimorbidity (19). It is worth mentioning that we found that the prevalence of multimorbidity increased more rapidly among populations with initial unhealthy lifestyles. A longitudinal study of lifestyle implied that health-risk behaviors tend to be stable as individuals age (19). These findings highlight the need to pay more attention to high-risk populations and strengthen interventions for modifiable behavioral factors in the early stages.

Cardiometabolic multimorbidity is the most often pattern that has been identified (7, 20). The present study found a high prevalence of the combinations of hypertension + diabetes, hypertension + stroke, and hypertension + CHD. Hypertension and diabetes are the main components of metabolic syndrome and share pathophysiological mechanisms (21). Hypertension is the primary risk factor for cardiovascular diseases such as CHD and stroke, making it likely that these conditions occur together. Chinese researchers discovered that lifestyle factors play different roles in varying stages of the cardiometabolic status in the CKB cohort (22). It suggests that lifestyle modifications at any point are beneficial in preventing and managing cardiometabolic multimorbidity. The Chinese CHERRY study utilized data from an electronic medical record system and found that the prevalence of cardiometabolic multimorbidity had more than doubled over the past 5 years, implying that the impact of multimorbidity needs to be considered in the primary and secondary prevention of cardiovascular diseases (23). The UK Biobank cohort study revealed an association of cardiometabolic multimorbidity with mortality from all causes, cardiovascular diseases, and cancer (24). In contrast, the present study did not find a connection between cardiometabolic multimorbidity and cancer mortality. This discrepancy may be related to differences in the prevalence of cancer within the study populations. Our observations indicated that the presence of cardiometabolic multimorbidity significantly increased the risk of diabetes mortality, with an HR of 28.19 (95% CI = 14.85–53.51), which has rarely been reported previously.

Respiratory multimorbidity has received considerable attention. According to the Global Burden of Disease Study, COPD is now the third leading cause of death after heart disease and stroke (25). Both COPD and asthma are common conditions characterized by chronic inflammatory reactions in the airway. Asthma patients who suffer persistent airflow restriction may progress to COPD and develop an asthma-COPD overlap syndrome (26). There is increasing evidence of a bidirectional relationship between COPD and tuberculosis, each of which could become an independent risk factor for the other (27). The results of the National Health Interview Survey conducted in the US showed that individuals with respiratory multimorbidity had a 2.14-fold higher risk of death compared with healthy individuals, a 13.65-fold higher risk of death from chronic lower respiratory diseases, and a 1.87-fold higher risk of cancer (20). However, no association between respiratory multimorbidity and cancer mortality was found in the present study.

In contrast to national results for mental and arthritis multimorbidity (7), we observed that kidney disease also tended to be associated with this pattern. UK researchers discovered that depression was prevalent among patients with CKD and explored the effect of integrating psychological management into routine kidney care (28). The World Mental Health Survey found an association between mental disorders and arthritis in 17 countries, indicating that the psychological strain caused by arthritis may trigger the onset of psychiatric disorders (29). A systematic review identified a multimorbidity pattern of mental health in all 16 studies (30), implying that the mental health status of patients should be carefully considered in clinical treatments and caregiving. A survey of the older adult population in the US showed that the neuropsychiatric class exhibited the highest mortality across several patterns of multimorbidity (31). Although our study found that the mortality risk was not higher in the presence of mental, kidney, and arthritis multimorbidity, it is essential to pay attention to clustering effects and be aware of potential growth trends and mortality risks.

The subgroup analyses revealved that age was a modifying factor in the relationships of multimorbidity with mortality from all causes, four major chronic diseases, and cancer. Consistent with findings from the UK Biobank study (24), we observed that the mortality risk due to multimorbidity was higher in the non-senior population, which may attributed to the high mortality rate in the older adult population at baseline and hence relatively low additional mortality rate caused by multimorbidity during the follow-up. This implied that the focus should be on young and middle-aged populations when attempting to prevent and manage multimorbidity, which may help to reduce premature death. This study also discovered that although the prevalence of multimorbidity was lower in married individuals (32), they had an elevated risk of cardiovascular disease death as a result of multimorbidity. Further studies should evaluate the effects of the quality, duration, and evolution of the marriage status and the influence of other social factors.

Several strengths distinguish this study. It had the advantages of a prospective research design and a long-term follow-up. We investigated temporal changes in multimorbidity prevalence and observed that high-risk individuals at baseline experienced a more rapid progression of multimorbidity during the follow-up, thereby exacerbating the initial situation, which has rarely been reported previously. In addition to overall mortality, this study also examined outcomes of four major chronic diseases, providing region-specific local evidence for preventing and controlling major chronic diseases in economically developed regions.

This study also had some limitations. First, the baseline information about chronic disease was collected through self-reporting, which may be subject to inadequate awareness or recall bias and lead to a reduced prevalence of baseline chronic diseases. Second, we may have missed asymptomatic patients or those with mild symptoms only treated in outpatient clinics, potentially resulting in an underestimation of prevalence rates based on information obtained from hospitalization in the health insurance system during the follow-up. Third, some combinations that did not significantly affect the mortality rate might have been due to the limited size of the cohort population and the number of deaths.

## 5 Conclusion

In conclusion, we explored the temporal change in the prevalence of multimorbidity in the Wuzhong district, identified common cluster patterns, and quantified their impact on mortality. In the context of aging and the associated increasing disease burden, this study has provided local evidence for chronic disease intervention strategies and health management in economically developed regions and highlighted the need to consider high-risk populations.

## Data availability statement

The data that support the findings of this study are available from the corresponding author upon reasonable request, with permission from the Department of China Kadoorie Biobank.

## Ethics statement

The studies involving humans were approved by the Ethics Review Committee of the Chinese Center for Disease Control and Prevention (Beijing, China) and the Oxford Tropical Research Ethics Committee, University of Oxford (UK). The studies were conducted in accordance with the local legislation and institutional requirements. The participants provided their written informed consent to participate in this study.

## Author contributions

HY: Formal analysis, Investigation, Methodology, Project administration, Software, Supervision, Writing – original draft, Writing – review & editing. RT: Investigation, Project administration, Supervision, Writing – review & editing. JZ: Investigation, Project administration, Supervision, Writing – review & editing. JS: Investigation, Project administration, Supervision, Writing – review & editing. YL: Data curation, Investigation, Supervision, Writing – review & editing. YH: Data curation, Investigation, Supervision, Writing – review & editing. JJ: Data curation, Investigation, Supervision, Writing – review & editing. PP: Data curation, Methodology, Project administration, Supervision, Writing – review & editing. CY: Conceptualization, Data curation, Methodology, Project administration, Software, Supervision, Validation, Writing – review & editing. DS: Data curation, Methodology, Project administration, Software, Validation, Writing – review & editing. ZC: Writing – review & editing, Funding acquisition, Project administration, Resources, Supervision. LL: Funding acquisition, Project administration, Resources, Supervision, Writing – review & editing. JL: Conceptualization, Data curation, Methodology, Project administration, Resources, Supervision, Validation, Visualization, Writing – review & editing.
