# Supplementary material for: Temporal change in multimorbidity prevalence, clustering patterns, and the association with mortality: findings from the China Kadoorie Biobank study in Jiangsu Province
Source: Front Public Health. 2024 Apr 18;12:1389635. doi: 10.3389/fpubh.2024.1389635 (PMC11064014; doi:10.3389/fpubh.2024.1389635)
Supplement: Supplementary file 1 [file Table_1.docx]

Supplementary Material

**Temporal change in multimorbidity prevalence, clustering patterns, and the association with mortality: findings from the China Kadoorie Biobank study in Jiangsu Province**

**Hao Yu^1,2^, Ran Tao^2^, Jinyi Zhou^2^, Jian Su^2^, Yan Lu^3^, Yujie Hua^3^, Jianrong Jin^4^, Pei Pei^5^, Canqing Yu^1,5,6^, Dianjianyi Sun^1,5,6^, Zhengming Chen^7^, Liming Li^1,5,6^, Jun Lv^1,5,6*^**

*** Correspondence:**Jun Lv

lvjun@bjmu.edu.cn

# Supplementary Tables

**Table S1** Prevalence at baseline and the end of follow-up by different demographic characteristics

| **Baseline characteristics** | **Baseline** | | | | | |  | **The end of follow-up** | | | | | | **Change (%)** |
| --- | --- | --- | --- | --- | --- | --- | --- | --- | --- | --- | --- | --- | --- | --- |
|  | **No. of participants** | **Prevalence（%）** | | | | **Multimorbidity**  **prevalence（%）** |  | **No. of participants** | **Prevalence（%）** | | | | **Multimorbidity**  **prevalence（%）** |  |
|  |  | **0-1** | **2** | **3** | **4** |  |  |  | 0-1 | 2 | 3 | 4 |  |  |
| All | 53269 | 78.9 | 15.6 | 4.4 | 1.1 | 21.1 |  | 49391 | 72.3 | 18.2 | 6.8 | 2.7 | 27.7 | 6.6 |
| Sex |  |  |  |  |  |  |  |  |  |  |  |  |  |  |
| male | 22366 | 78.9 | 16.1 | 4.0 | 1.0 | 21.1 |  | 20240 | 72.2 | 18.7 | 6.6 | 2.5 | 27.7 | 6.6 |
| female | 30903 | 78.9 | 15.3 | 4.6 | 1.2 | 21.1 |  | 29151 | 72.2 | 17.9 | 7.0 | 2.9 | 27.8 | 6.7 |
| Age, year |  |  |  |  |  |  |  |  |  |  |  |  |  |  |
| <60 | 40605 | 84.5 | 12.2 | 2.8 | 0.5 | 15.5 |  | 39216 | 78.4 | 15.4 | 4.8 | 1.4 | 21.5 | 6.0 |
| ≥60 | 12664 | 61.2 | 26.5 | 9.2 | 3.1 | 38.9^*^ |  | 10175 | 48.4 | 29.0 | 14.7 | 7.9 | 51.6^*^ | 12.7 |
| Education level | | |  |  |  |  |  |  |  |  |  |  |  |  |
| primary school and below | 33175 | 76.1 | 17.6 | 5.0 | 1.3 | 23.9 |  | 30236 | 68.0 | 20.5 | 8.1 | 3.4 | 32.0 | 8.1 |
| middle school and above | 20094 | 83.6 | 12.3 | 3.2 | 0.9 | 16.4^*^ |  | 19155 | 79.0 | 14.6 | 4.8 | 1.6 | 21.0^*^ | 4.6 |
| Marital status |  |  |  |  |  |  |  |  |  |  |  |  |  |  |
| married | 49395 | 79.8 | 15.1 | 4.1 | 1.0 | 20.3 |  | 46139 | 73.1 | 17.7 | 6.6 | 2.6 | 26.9 | 6.6 |
| unmarried | 3874 | 69.0 | 21.7 | 7.2 | 2.1 | 31.0^*^ |  | 3252 | 59.8 | 24.8 | 10.4 | 5.0 | 40.1^*^ | 9.1 |
| Occupation |  |  |  |  |  |  |  |  |  |  |  |  |  |  |
| workers and farmers | 20015 | 85.5 | 11.8 | 2.4 | 0.3 | 14.5 |  | 18972 | 79.5 | 14.9 | 4.4 | 1.2 | 20.6 | 6.1 |
| others | 33254 | 75.0 | 17.9 | 5.5 | 1.6 | 25.0^*^ |  | 30419 | 67.9 | 20.2 | 8.3 | 3.6 | 32.2^*^ | 7.2 |
| Household income, yuan | | |  |  |  |  |  |  |  |  |  |  |  |  |
| <20000 | 14196 | 71.6 | 20.5 | 6.2 | 1.7 | 28.4 |  | 12255 | 62.5 | 23.0 | 9.9 | 4.6 | 37.5 | 9.1 |
| ≥20000 | 39073 | 81.6 | 13.8 | 3.7 | 0.9 | 18.4^*^ |  | 37136 | 75.5 | 16.6 | 5.8 | 2.1 | 24.5^*^ | 6.1 |
| Smoke status, % |  |  |  |  |  |  |  |  |  |  |  |  |  |  |
| not smokers | 34892 | 78.8 | 15.4 | 4.6 | 1.2 | 21.2 |  | 32810 | 72.1 | 18.1 | 7.0 | 2.8 | 27.9 | 6.7 |
| daily smokers | 18377 | 79.1 | 15.9 | 4.0 | 1.0 | 20.8 |  | 16581 | 72.6 | 18.5 | 6.4 | 2.5 | 27.4 | 6.6 |
| Alcohol consumption, % | |  |  |  |  |  |  |  |  |  |  |  |  |  |
| non-excessive drinkers | 45666 | 79.5 | 15.1 | 4.3 | 1.1 | 20.5 |  | 42653 | 72.9 | 17.7 | 6.7 | 2.7 | 27.0 | 6.5 |
| excessive drinkers | 7603 | 75.8 | 18.4 | 4.7 | 1.1 | 24.2^*^ |  | 6738 | 67.8 | 21.4 | 7.9 | 2.9 | 32.1^*^ | 7.9 |
| Physical activity | |  |  |  |  |  |  |  |  |  |  |  |  |  |
| low | 17723 | 68.0 | 22.0 | 7.5 | 2.5 | 31.9 |  | 15529 | 60.4 | 23.2 | 10.9 | 5.5 | 39.6 | 7.7 |
| middle | 17787 | 82.1 | 13.8 | 3.5 | 0.6 | 17.9^*^ |  | 16785 | 75.0 | 17.3 | 5.8 | 1.9 | 24.9^*^ | 7.0 |
| high | 17759 | 86.5 | 11.0 | 2.2 | 0.3 | 13.5^*^ |  | 17077 | 80.4 | 14.5 | 4.1 | 1.0 | 19.7^*^ | 6.2 |
| Vegetables consumption | |  |  |  |  |  |  |  |  |  |  |  |  |  |
| not frequent | 217 | 74.2 | 18.0 | 7.8 | 0.0 | 25.8 |  | 191 | 64.9 | 23.6 | 10.5 | 1.0 | 35.1 | 9.3 |
| frequent | 53052 | 79.0 | 15.6 | 4.3 | 1.1 | 21.0^*^ |  | 49200 | 72.3 | 18.2 | 6.8 | 2.7 | 27.7^*^ | 6.7 |
| Fruits consumption | |  |  |  |  |  |  |  |  |  |  |  |  |  |
| not frequent | 37922 | 78.5 | 16.0 | 4.4 | 1.1 | 21.4 |  | 35031 | 71.6 | 18.7 | 6.9 | 2.8 | 28.5 | 7.1 |
| frequent | 15347 | 79.8 | 14.7 | 4.2 | 1.3 | 20.1^*^ |  | 14360 | 74.1 | 16.9 | 6.5 | 2.5 | 25.9^*^ | 5.8 |
| Meat consumption | |  |  |  |  |  |  |  |  |  |  |  |  |  |
| not frequent | 29483 | 76.0 | 17.3 | 5.2 | 1.5 | 24.0 |  | 26919 | 69.1 | 19.6 | 7.8 | 3.5 | 31.0 | 7.0 |
| frequent | 23786 | 82.5 | 13.5 | 3.3 | 0.7 | 17.4^*^ |  | 22472 | 76.2 | 16.5 | 5.6 | 1.7 | 23.9^*^ | 6.5 |
| Overweight or obese | |  |  |  |  |  |  |  |  |  |  |  |  |  |
| no | 24632 | 82.7 | 13.1 | 3.4 | 0.8 | 17.3 |  | 22575 | 78.5 | 14.7 | 5.0 | 1.8 | 21.6 | 4.3 |
| yes | 28637 | 75.6 | 17.8 | 5.2 | 1.4 | 24.3^*^ |  | 26816 | 67.1 | 21.1 | 8.3 | 3.5 | 32.9^*^ | 8.6 |
| Family history of chronic diseases | |  |  |  |  |  |  |  |  |  |  |  |  |  |
| no | 29114 | 81.2 | 14.4 | 3.6 | 0.8 | 18.8 |  | 26947 |  | 16.7 | 5.9 | 2.2 | 24.8 | 6.0 |
| yes | 24155 | 76.3 | 17.0 | 5.2 | 1.5 | 23.7^*^ |  | 22444 |  | 20.0 | 7.9 | 3.3 | 31.3^*^ | 7.6 |

^*^ There was a statistical difference between the groups (P＜0.05), and the reference group was the first row of each factor.

Marital status: unmarried including widowed, separated or divorced; smoke status: not smokers included non-smokers or seldom smokers, daily smokers included current smokers and former smokers; alcohol consumption: non-excessive drinkers included non-drinkers, seldom drinkers or daily consumption <30 grams, excessive drinkers included stop drinking or daily consumption ≥30 grams), physical activity (MET h/d, trisected into low, middle, high groups), fresh vegetables, fruits, and meat consumption frequent: consume at least four times a week; overweight or obesity: BMI ≥ 24kg/m^2^, WC ≥85cm for males, or ≥80cm for females.

**Table S2** Association between baseline cardiometabolic combinations and mortality

| **Combinations^a^** | | **No. of**  **multimorbidity** | **No. of deaths** | **Mortality^b^** | **Model 1**  **HR(95%CI)^c^** | **Model 2**  **HR (95%CI)^c^** | **Model 3**  **HR (95%CI)^c^** |
| --- | --- | --- | --- | --- | --- | --- | --- |
| All causes | |  |  |  |  |  |  |
|  | Non-cardiometabolic multimorbidity | 50745 | 3008 | 5.42 | 1.00 | 1.00 | 1.00 |
|  | Hypertension + diabetes | 1719 | 232 | 12.98 | 1.54 (1.35-1.76) | 1.64 (1.43-1.88) | 1.59 (1.39-1.82) |
|  | Hypertension + stroke | 331 | 85 | 25.75 | 2.04 (1.64-2.53) | 1.86 (1.50-2.32) | 1.77 (1.42-2.20) |
|  | Hypertension + CHD | 313 | 62 | 18.78 | 1.35 (1.05-1.74) | 1.39 (1.08-1.79) | 1.39 (1.08-1.80) |
|  | Hypertension + stroke + diabetes | 62 | 17 | 29.93 | 2.63 (1.63-4.24) | 2.63 (1.63-4.25) | 2.65 (1.64-4.29) |
|  | Hypertension + CHD + diabetes | 61 | 16 | 26.03 | 1.78 (1.09-2.92) | 2.11 (1.29-3.45) | 1.94 (1.18-3.19) |
|  | Hypertension + stroke + CHD | 17 | 7 | 45.40 | 2.20 (1.05-4.62) | 1.83 (0.87-3.86) | 1.66 (0.79-3.51) |
| Four major chronic diseases | |  |  |  |  |  |  |
|  | Non-cardiometabolic multimorbidity | 50745 | 2489 | 4.48 | 1.00 | 1.00 | 1.00 |
|  | Hypertension + diabetes | 1719 | 197 | 11.03 | 1.55 (1.34-1.80) | 1.66 (1.44-1.93) | 1.61 (1.39-1.87) |
|  | Hypertension + stroke | 331 | 74 | 22.42 | 2.09 (1.66-2.64) | 1.92 (1.52-2.42) | 1.83 (1.45-2.31) |
|  | Hypertension + CHD | 313 | 52 | 15.75 | 1.34 (1.02-1.76) | 1.38 (1.05-1.82) | 1.39 (1.05-1.83) |
|  | Hypertension + stroke + diabetes | 62 | 17 | 29.93 | 3.08 (1.91-4.97) | 3.09 (1.92-5.00) | 3.13 (1.93-5.06) |
|  | Hypertension + CHD + diabetes | 61 | 14 | 22.78 | 1.84 (1.09-3.11) | 2.20 (1.30-3.73) | 2.01 (1.18-3.41) |
|  | Hypertension + stroke + CHD | 17 | 7 | 45.40 | 2.57 (1.22-5.39) | 2.11 (1.00-4.45) | 1.91 (0.90-4.04) |
| Cardiovascular diseases | |  |  |  |  |  |  |
|  | Non-cardiometabolic multimorbidity | 50745 | 736 | 1.33 | 1.00 | 1.00 | 1.00 |
|  | Hypertension + diabetes | 1719 | 61 | 3.41 | 1.58 (1.22-2.05) | 1.69 (1.30-2.20) | 1.63 (1.25-2.12) |
|  | Hypertension + stroke | 331 | 54 | 16.36 | 4.55 (3.45-6.01) | 4.33 (3.27-5.74) | 4.04 (3.03-5.37) |
|  | Hypertension + CHD | 313 | 31 | 9.39 | 2.33 (1.63-3.34) | 2.40 (1.67-3.44) | 2.36 (1.64-3.39) |
|  | Hypertension + stroke + diabetes | 62 | 11 | 19.37 | 6.39 (3.52-11.61) | 6.74 (3.69-12.30) | 6.47 (3.53-11.88) |
|  | Hypertension + CHD + diabetes | 61 | 8 | 13.02 | 2.98 (1.48-5.99) | 3.61 (1.78-7.29) | 3.30 (1.63-6.69) |
|  | Hypertension + stroke + CHD | 17 | 5 | 32.43 | 5.07 (2.10-12.24) | 4.23 (1.73-10.32) | 3.81 (1.55-9.35) |
| Diabetes | |  |  |  |  |  |  |
|  | Non-cardiometabolic multimorbidity | 50745 | 22 | 0.04 | 1.00 | 1.00 | 1.00 |
|  | Hypertension + diabetes | 1719 | 40 | 2.24 | 38.34 (22.49-65.35) | 45.50 (26.52-78.07) | 43.78 (24.93-76.88) |

HR: Hazard ratios; CI: Confidence intervals; CHD: Coronary heart disease.

^a^The results with a combination frequency or corresponding deaths less than 5 were excluded and not reported in the table. ^b^The mortality was calculated by dividing the number of deaths by the number of follow-up years and multiplying by 1000. ^c^ Multivariable models were adjusted for the same covariates as those in Table 2.

**Table S3** Association between baseline respiratory combinations and mortality

| **Combinations^a^** | | **No. of**  **multimorbidity** | **No. of deaths** | **Mortality** | **Model 1**  **HR(95%CI)** | **Model 2**  **HR (95%CI)** | **Model 3**  **HR (95%CI)** |
| --- | --- | --- | --- | --- | --- | --- | --- |
| All causes | |  |  |  |  |  |  |
|  | Non-respiratory multimorbidity | 52895 | 3348 | 5.80 | 1.00 | 1.00 | 1.00 |
|  | COPD + asthma | 231 | 47 | 19.57 | 1.77 (1.32-2.35) | 1.60 (1.20-2.13) | 1.56 (1.17-2.09) |
|  | COPD + tuberculosis | 134 | 33 | 24.55 | 1.96 (1.39-2.76) | 1.66 (1.18-2.34) | 1.65 (1.17-2.33) |
|  | COPD + asthma + tuberculosis | 9 | 5 | 56.37 | 4.20 (1.75-10.10) | 2.88 (1.20-6.94) | 2.72 (1.13-6.57) |
| Four major chronic diseases | |  |  |  |  |  |  |
|  | Non-respiratory multimorbidity | 52895 | 2779 | 4.81 | 1.00 | 1.00 | 1.00 |
|  | COPD + asthma | 231 | 44 | 18.32 | 1.96 (1.45-2.64) | 1.77 (1.31-2.39) | 1.72 (1.27-2.33) |
|  | COPD + tuberculosis | 134 | 28 | 20.83 | 1.95 (1.34-2.83) | 1.64 (1.13-2.38) | 1.62 (1.11-2.36) |
| Respiratory diseases | |  |  |  |  |  |  |
|  | Non-respiratory multimorbidity | 52895 | 190 | 0.33 | 1.00 | 1.00 | 1.00 |
|  | COPD + asthma | 231 | 21 | 8.74 | 11.50 (7.31-18.09) | 9.46 (5.93-15.10) | 9.23 (5.71-14.92) |
|  | COPD + tuberculosis | 134 | 5 | 3.72 | 4.41 (1.81-10.73) | 3.51 (1.43-8.62) | 3.43 (1.39-8.45) |

HR: Hazard ratios; CI: Confidence intervals; COPD: Chronic obstructive pulmonary disease.

Models were adjusted for the same covariates as those in Table 2.

**Table S4** Sensitivity analysis of the association between baseline chronic disease numbers and mortality with additional adjustments for BMI and WC

| **Causes** | **No. of chronic diseases** | **No. of deaths** | **Mortality^a^** | **HR (95%CI)^b^** |
| --- | --- | --- | --- | --- |
| All causes | |  |  |  |
|  | 0-1 | 2020 | 4.37 | 1.00 |
|  | ≥2 | 1413 | 11.84 | 1.43 (1.33-1.54) |
|  | trend for numbers | | | 1.26 (1.21-1.31) |
| Four major chronic diseases | | |  |  |
|  | 0-1 | 1667 | 3.61 | 1.00 |
|  | ≥2 | 1188 | 9.95 | 1.42 (1.31-1.54) |
|  | trend for numbers | | | 1.27 (1.22-1.33) |

HR: Hazard ratios; CI: Confidence intervals.

^†^ The mortality increased with the number of chronic diseases.

^a^The mortality was calculated by dividing the number of deaths by the number of follow-up years and multiplying by 1000. ^b^The covariates were the same as Model 3 in Table 2, with additional adjustments for BMI and WC.

**Table S5** Sensitivity analysis of the association between baseline multimorbidity patterns and mortality with additional adjustments for BMI and WC

| **Causes** | **Patterns** | **No. of multimorbidity** | **No. of death** | **Mortality^a^** | **HR (95%CI)^b^** |
| --- | --- | --- | --- | --- | --- |
| All causes | |  |  |  |  |
|  | Non multimorbidity | 42047 | 2020 | 4.37 | 1.00 |
|  | Cardiometabolic multimorbidity | 2524 | 425 | 16.34 | 1.89 (1.69-2.12) |
|  | Respiratory multimorbidity | 374 | 85 | 22.17 | 1.79 (1.43-2.23) |
|  | Mental, kidney and arthritis multimorbidity | 279 | 20 | 6.50 | 1.23 (0.79-1.91) |
| Four maojr chronic diseases | |  |  |  |  |
|  | Non multimorbidity | 42047 | 1667 | 3.61 | 1.00 |
|  | Cardiometabolic multimorbidity | 2524 | 366 | 14.07 | 1.94 (1.71-2.19) |
|  | Respiratory multimorbidity | 374 | 76 | 19.82 | 1.85 (1.46-2.34) |
|  | Mental, kidney and arthritis multimorbidity | 279 | 14 | 4.55 | 1.03 (0.61-1.75) |

HR: Hazard ratios; CI: Confidence intervals.

^a^The mortality was calculated by dividing the number of deaths by the number of follow-up years and multiplying by 1000. ^b^The covariates were the same as Model 3 in table 2, with additional adjustments for BMI and WC.

**Table S6** Sensitivity analysis of the association between baseline multimorbidity combinations and all-cause mortality with additional adjustments for BMI and WC

| **Combinations** | | **No. of multimorbidity** | **All causes mortality^a^** | **Four major chronic diseases mortality** | **All causes HR(95% CI)^b^** | **Four major chronic diseases**  **HR(95% CI)^b^** |
| --- | --- | --- | --- | --- | --- | --- |
| Cardiometabolic multimorbidity | | |  |  |  |  |
|  | Non-cardiometabolic multimorbidity | 50745 | 5.42 | 4.48 | 1.00 | 1.00 |
|  | Hypertension + diabetes | 1719 | 12.98 | 11.03 | 1.69 (1.48-1.94) | 1.70 (1.47-1.98) |
|  | Hypertension + stroke | 331 | 25.75 | 22.42 | 1.86 (1.49-2.31) | 1.91 (1.51-2.42) |
|  | Hypertension + CHD | 313 | 18.78 | 15.75 | 1.45 (1.12-1.87) | 1.44 (1.09-1.90) |
|  | Hypertension + stroke + diabetes | 62 | 29.93 | 29.93 | 2.89 (1.78-4.67) | 3.36 (2.08-5.45) |
|  | Hypertension + CHD + diabetes | 61 | 26.03 | 22.78 | 2.09 (1.27-3.43) | 2.14 (1.26-3.64) |
|  | Hypertension + stroke + CHD | 17 | 45.40 | 45.40 | 1.64 (0.78-3.47) | 1.88 (0.89-3.97) |
| Respiratory multimorbidity | |  |  |  |  |  |
|  | Non-respiratory multimorbidity | 52895 | 5.80 | 4.81 | 1.00 | 1.00 |
|  | COPD + asthma | 231 | 19.57 | 18.32 | 1.57 (1.17-2.10) | 1.72 (1.27-2.32) |
|  | COPD + tuberculosis | 134 | 24.55 | 20.83 | 1.58 (1.12-2.23) | 1.55 (1.07-2.26) |

HR: Hazard ratios; CI: Confidence intervals; CHD: Coronary heart disease; COPD: Chronic obstructive pulmonary disease.

^a^The mortality was calculated by dividing the number of deaths by the number of follow-up years and multiplying by 1000. ^b^The covariates were the same as Model 3 in Table 2, with additional adjustments for BMI and WC.

**Table S7** Subgroup analysis of the association between baseline multimorbidity status and the all-cause mortality

| **Baseline characteristics** | |  | **Deaths of non-multimorbidity** | |  | **Deaths of multimorbidity** | |  | **P_interaction_** |
| --- | --- | --- | --- | --- | --- | --- | --- | --- | --- |
|  |  |  | **No. of cases** | **HR** |  | **No. of cases** | **HR(95%CI)** |  |  |
| Sex | |  |  |  |  |  |  |  | 0.417 |
|  | male |  | 1172 | 1.00 |  | 799 | 1.38 (1.25-1.52) |  |  |
|  | female |  | 848 | 1.00 |  | 614 | 1.45 (1.30-1.62) |  |  |
| Age, year | |  |  |  |  |  |  |  | ＜0.001 |
|  | ＜60 |  | 759 | 1.00 |  | 325 | 1.75 (1.53-2.00) |  |  |
|  | ≥60 |  | 1261 | 1.00 |  | 1088 | 1.28 (1.18-1.39) |  |  |
| Education level | |  |  |  |  |  |  |  | 0.429 |
|  | primary school and below |  | 1626 | 1.00 |  | 1149 | 1.39 (1.28-1.50) |  |  |
|  | middle school and above |  | 394 | 1.00 |  | 264 | 1.53 (1.29-1.82) |  |  |
| Marital status | |  |  |  |  |  |  |  | 0.705 |
|  | married |  | 1699 | 1.00 |  | 1144 | 1.42 (1.31-1.54) |  |  |
|  | unmarried |  | 321 | 1.00 |  | 269 | 1.36 (1.15-1.61) |  |  |
| Smoking status,% | |  |  |  |  |  |  |  | 0.851 |
|  | not smokers |  | 1021 | 1.00 |  | 728 | 1.42 (1.28-1.56) |  |  |
|  | daily smokers |  | 999 | 1.00 |  | 685 | 1.41 (1.27-1.56) |  |  |
| Alcohol consumption,% | | |  |  |  |  |  |  | 0.596 |
|  | non-excessive drinkers |  | 1549 | 1.00 |  | 1068 | 1.42 (1.31-1.54) |  |  |
|  | excessive drinkers |  | 471 | 1.00 |  | 345 | 1.38 (1.19-1.60) |  |  |
| Physical activity | |  |  |  |  |  |  |  | 0.812 |
|  | low |  | 998 | 1.00 |  | 966 | 1.39 (1.27-1.52) |  |  |
|  | middle |  | 577 | 1.00 |  | 292 | 1.45 (1.25-1.68) |  |  |
|  | high |  | 445 | 1.00 |  | 155 | 1.49 (1.23-1.79) |  |  |
| Female menopausal status | | | |  |  |  |  |  | 0.758 |
|  | premenopausal |  | 137 | 1.00 |  | 28 | 1.43 (0.94-2.17) |  |  |
|  | menopausal |  | 710 | 1.00 |  | 586 | 1.45 (1.29-1.62) |  |  |
| Overweight or obese | |  |  |  |  |  |  |  | 0.942 |
|  | no |  | 1148 | 1.00 |  | 710 | 1.42 (1.29-1.56) |  |  |
|  | yes |  | 872 | 1.00 |  | 703 | 1.44 (1.30-1.60) |  |  |
| Family history of chronic diseases | | | | |  |  |  |  | 0.203 |
|  | no |  | 1191 | 1.00 |  | 746 | 1.35 (1.23-1.49) |  |  |
|  | yes |  | 829 | 1.00 |  | 667 | 1.49 (1.34-1.66) |  |  |

HR: Hazard ratios; CI: Confidence intervals.

Marital status: unmarried including widowed, separated or divorced; smoke status: not smokers included non-smokers or seldom smokers, daily smokers included current smokers and former smokers; alcohol consumption: non-excessive drinkers included non-drinkers, seldom drinkers or daily consumption ＜30 grams, excessive drinkers included stop drinking or daily consumption ≥30 grams), physical activity (MET h/d, trisected into low, middle, high groups), overweight or obesity: BMI ≥ 24kg/m2, WC ≥ 85cm/male, or 80cm/ female.

**Table S8** Subgroup analysis of the association between baseline multimorbidity status and mortality from four major chronic diseases

| **Baseline characteristics** | |  | **Deaths of non-multimorbidity** | |  | **Deaths of multimorbidity** | |  | **P_interaction_** |
| --- | --- | --- | --- | --- | --- | --- | --- | --- | --- |
|  |  |  | **No. of cases** | **HR** |  | **No. of cases** | **HR(95%CI)** |  |  |
| Sex | |  |  |  |  |  |  |  | 0.272 |
|  | male |  | 990 | 1.00 |  | 681 | 1.34 (1.21-1.48) |  |  |
|  | female |  | 677 | 1.00 |  | 507 | 1.49 (1.32-1.68) |  |  |
| Age, year | |  |  |  |  |  |  |  | 0.002 |
|  | ＜60 |  | 598 | 1.00 |  | 254 | 1.67 (1.44-1.95) |  |  |
|  | ≥60 |  | 1069 | 1.00 |  | 934 | 1.30 (1.19-1.43) |  |  |
| Education level | |  |  |  |  |  |  |  | 0.578 |
|  | primary school and below |  | 1356 | 1.00 |  | 970 | 1.38 (1.27-1.51) |  |  |
|  | middle school and above |  | 311 | 1.00 |  | 218 | 1.48 (1.22-1.79) |  |  |
| Marital status | |  |  |  |  |  |  |  | 0.556 |
|  | married |  | 1402 | 1.00 |  | 970 | 1.42 (1.30-1.55) |  |  |
|  | unmarried |  | 265 | 1.00 |  | 218 | 1.33 (1.11-1.61) |  |  |
| Smoking status,% | |  |  |  |  |  |  |  | 0814 |
|  | not smokers |  | 820 | 1.00 |  | 596 | 1.43 (1.28-1.60) |  |  |
|  | daily smokers |  | 847 | 1.00 |  | 592 | 1.38 (1.24-1.55) |  |  |
| Alcohol consumption,% | |  |  |  |  |  |  |  | 0.888 |
|  | non-excessive drinkers |  | 1268 | 1.00 |  | 885 | 1.40 (1.28-1.54) |  |  |
|  | excessive drinkers |  | 399 | 1.00 |  | 303 | 1.39 (1.19-1.63) |  |  |
| Physical activity | |  |  |  |  |  |  |  | 0.864 |
|  | low |  | 829 | 1.00 |  | 815 | 1.39 (1.26-1.53) |  |  |
|  | middle |  | 475 | 1.00 |  | 242 | 1.42 (1.21-1.67) |  |  |
|  | high |  | 363 | 1.00 |  | 131 | 1.47 (1.20-1.80) |  |  |
| Female menopausal status | | | |  |  |  |  |  | 0.584 |
|  | premenopausal |  | 107 | 1.00 |  | 22 | 1.38 (0.86-2.20) |  |  |
|  | menopausal |  | 569 | 1.00 |  | 485 | 1.49 (1.32-1.69) |  |  |
| Overweight or obese | |  |  |  |  |  |  |  | 0.552 |
|  | no |  | 959 | 1.00 |  | 595 | 1.38 (1.24-1.53) |  |  |
|  | yes |  | 708 | 1.00 |  | 593 | 1.47 (1.31-1.65) |  |  |
| Family history of chronic diseases | | | |  |  |  |  |  | 0.091 |
|  | no |  | 979 | 1.00 |  | 614 | 1.32 (1.19-1.46) |  |  |
|  | yes |  | 688 | 1.00 |  | 574 | 1.52 (1.35-1.71) |  |  |

HR: Hazard ratios; CI: Confidence intervals.

The definitions of categorical variables were the same as in Supplementary Table 7.

**Table S9** Subgroup analysis of the association between baseline multimorbidity status and mortality from cardiovascular disease

| **Baseline characteristics** | |  | **Deaths of non-multimorbidity** | |  | **Deaths of multimorbidity** | |  | **P_interaction_** |
| --- | --- | --- | --- | --- | --- | --- | --- | --- | --- |
|  |  |  | **No. of cases** | **HR** |  | **No. of cases** | **HR(95%CI)** |  |  |
| Sex | |  |  |  |  |  |  |  | 0.867 |
|  | male |  | 256 | 1.00 |  | 232 | 1.52 (1.26-1.83) |  |  |
|  | female |  | 224 | 1.00 |  | 198 | 1.53 (1.25-1.86) |  |  |
| Age, year | |  |  |  |  |  |  |  | 0.241 |
|  | ＜60 |  | 109 | 1.00 |  | 52 | 1.78 (1.27-2.51) |  |  |
|  | ≥60 |  | 371 | 1.00 |  | 378 | 1.46 (1.26-1.69) |  |  |
| Education level | |  |  |  |  |  |  |  | 0.093 |
|  | primary school and below |  | 412 | 1.00 |  | 351 | 1.46 (1.26-1.69) |  |  |
|  | middle school and above |  | 68 | 1.00 |  | 79 | 2.01 (1.41-2.87) |  |  |
| Marital status | |  |  |  |  |  |  |  | 0.039 |
|  | married |  | 371 | 1.00 |  | 347 | 1.65 (1.42-1.92) |  |  |
|  | unmarried |  | 109 | 1.00 |  | 83 | 1.15 (0.86-1.55) |  |  |
| Smoking status,% | |  |  |  |  |  |  |  | 0.669 |
|  | not smokers |  | 273 | 1.00 |  | 236 | 1.48 (1.24-1.77) |  |  |
|  | daily smokers |  | 207 | 1.00 |  | 194 | 1.60 (1.30-1.97) |  |  |
| Alcohol consumption,% | | | |  |  |  |  |  | 0.713 |
|  | non-excessive drinkers |  | 381 | 1.00 |  | 328 | 1.53 (1.31-1.78) |  |  |
|  | excessive drinkers |  | 99 | 1.00 |  | 102 | 1.59 (1.19-2.13) |  |  |
| Physical activity | |  |  |  |  |  |  |  | 0.528 |
|  | low |  | 282 | 1.00 |  | 309 | 1.47 (1.24-1.73) |  |  |
|  | middle |  | 116 | 1.00 |  | 82 | 1.64 (1.22-2.20) |  |  |
|  | high |  | 82 | 1.00 |  | 39 | 1.74 (1.17-2.58) |  |  |
| Female menopausal status | | |  |  |  |  |  |  | 0.388 |
|  | premenopausal |  | 15 | 1.00 |  | 2 | 0.60 (0.13-2.80) |  |  |
|  | menopausal |  | 209 | 1.00 |  | 196 | 1.56 (1.28-1.90) |  |  |
| Overweight or obese | |  |  |  |  |  |  |  | 0.313 |
|  | no |  | 275 | 1.00 |  | 204 | 1.43 (1.18-1.72) |  |  |
|  | yes |  | 205 | 1.00 |  | 226 | 1.69 (1.38-2.06) |  |  |
| Family history of cardiovascular disease | | | | |  |  |  |  | 0.336 |
|  | no |  | 389 | 1.00 |  | 319 | 1.46 (1.26-1.71) |  |  |
|  | yes |  | 91 | 1.00 |  | 111 | 1.83 (1.36-2.45) |  |  |

HR: Hazard ratios; CI: Confidence intervals.

The definitions of categorical variables were the same as in Supplementary Table 7.

**Table S10** Subgroup analysis of the association between baseline multimorbidity status and cancer mortality

| **Baseline characteristics** | |  | **Deaths of non-multimorbidity** | |  | **Deaths of multimorbidity** | |  | **P_interaciont_** |
| --- | --- | --- | --- | --- | --- | --- | --- | --- | --- |
|  |  |  | **No. of cases** | **HR** |  | **No. of cases** | **HR(95%CI)** |  |  |
| Sex | |  |  |  |  |  |  |  | 0.561 |
|  | male |  | 662 | 1.00 |  | 355 | 1.14 (1.00-1.31) |  |  |
|  | female |  | 422 | 1.00 |  | 248 | 1.30 (1.10-1.53) |  |  |
| Age, year | |  |  |  |  |  |  |  | ＜0.001 |
|  | ＜60 |  | 469 | 1.00 |  | 180 | 1.54 (1.29-1.83) |  |  |
|  | ≥60 |  | 615 | 1.00 |  | 423 | 1.06 (0.93-1.20) |  |  |
| Education level | |  |  |  |  |  |  |  | 0.979 |
|  | primary school and below |  | 852 | 1.00 |  | 482 | 1.19 (1.06-1.34) |  |  |
|  | middle school and above |  | 232 | 1.00 |  | 121 | 1.22 (0.96-1.55) |  |  |
| Marital status | |  |  |  |  |  |  |  | 0.545 |
|  | married |  | 944 | 1.00 |  | 496 | 1.19 (1.06-1.33) |  |  |
|  | unmarried |  | 140 | 1.00 |  | 107 | 1.33 (1.02-1.73) |  |  |
| Smoking status,% | |  |  |  |  |  |  |  | 0.676 |
|  | not smokers |  | 512 | 1.00 |  | 286 | 1.21 (1.04-1.41) |  |  |
|  | daily smokers |  | 572 | 1.00 |  | 317 | 1.20 (1.04-1.38) |  |  |
| Alcohol consumption,% | |  |  |  |  |  |  |  | 0.474 |
|  | non-excessive drinkers |  | 806 | 1.00 |  | 448 | 1.22 (1.08-1.38) |  |  |
|  | excessive drinkers |  | 278 | 1.00 |  | 155 | 1.12 (0.91-1.37) |  |  |
| Physical activity | |  |  |  |  |  |  |  | 0.644 |
|  | low |  | 477 | 1.00 |  | 389 | 1.20 (1.05-1.38) |  |  |
|  | middle |  | 343 | 1.00 |  | 133 | 1.17 (0.95-1.43) |  |  |
|  | high |  | 264 | 1.00 |  | 81 | 1.30 (1.01-1.67) |  |  |
| Female menopausal status | | | |  |  |  |  |  | 0.848 |
|  | premenopausal |  | 90 | 1.00 |  | 19 | 1.44 (0.87-2.38) |  |  |
|  | menopausal |  | 331 | 1.00 |  | 229 | 1.28 (1.08-1.52) |  |  |
| Overweight or obese | |  |  |  |  |  |  |  | 0.890 |
|  | no |  | 607 | 1.00 |  | 295 | 1.19 (1.03-1.37) |  |  |
|  | yes |  | 477 | 1.00 |  | 308 | 1.24 (1.07-1.44) |  |  |
| Family history of cancer | | |  |  |  |  |  |  | 0.584 |
|  | no |  | 774 | 1.00 |  | 433 | 1.21 (1.07-1.37) |  |  |
|  | yes |  | 310 | 1.00 |  | 170 | 1.18 (0.97-1.43) |  |  |

HR: Hazard ratios; CI: Confidence intervals.

The definitions of categorical variables were the same as in Supplementary Table 7.
